# Pressure-based finite element analysis of setup distance in clear aligner canine retraction comparing bodily and tipping movements

**DOI:** 10.1038/s41598-026-51497-9

**Published:** 2026-04-30

**Authors:** Kiyean Kim, Youn-Kyung Choi, Sung-Hun Kim, Seong-Sik Kim, Yong-Il Kim

**Affiliations:** 1 Dental and Life Science Institute, School of Dentistry, Pusan National University, Yangsan, 50612 South Korea; 2 Department of orthodontics, dental Research Institute, School of Dentistry, Pusan National University, Yangsan, 50612 South Korea; 3 Department of Orthodontics, Dental and Life Science Institute, Dental Research Institute, School of Dentistry, Pusan National University, Geumoro 20, Mulgeumeup, 50612 Yangsan, South Korea

**Keywords:** Orthodontic biomechanics, Finite element analysis, Setup distance, Clear aligner therapy, Alveolar bone remodeling, Periodontal ligament, Anatomy, Engineering

## Abstract

In clear aligner therapy, setup distance is a critical design parameter governing orthodontic force magnitude, yet it is commonly determined empirically without explicit biomechanical justification. This study proposes a pressure-based biomechanical framework for evaluating setup distance during maxillary canine retraction. Three-dimensional finite element models were constructed with setup distances ranging from 0.05 to 0.35 mm, and both bodily and tipping movement designs were analyzed. Hydrostatic pressure distribution within the periodontal ligament (PDL) was calculated, and the area fraction within the physiological pressure range was quantified as a spatial metric associated with mechanically favorable conditions for bone remodeling. As setup distance increased, the proportion of PDL within the physiological pressure range initially increased and subsequently decreased as suprasystolic pressure regions expanded. Bodily movement exhibited a broader effective setup-distance range (0.10–0.25 mm) characterized by relatively uniform pressure distribution, whereas tipping movement showed a narrower effective range centered around 0.25 mm due to pronounced cervico–apical pressure gradients limiting expansion of the optimal pressure zone. These findings demonstrate that PDL pressure distribution provides a biomechanical basis for interpreting setup-distance selection in clear aligner design.

**Supplementary Information:**

The online version contains supplementary material available at 10.1038/s41598-026-51497-9.

## Introduction

Clear aligner therapy has become widely adopted as an alternative to fixed orthodontic appliances^1^ due to its aesthetic advantages^2^, improved patient comfort^3^, and compatibility with digital treatment planning^4^. Despite these benefits, achieving predictable tooth movement with clear aligners remains challenging, particularly for movements requiring precise biomechanical control, such as upper canine retraction^5–7^. In this study, setup distance is defined as the planned positional discrepancy between the current tooth position and the target position embedded in the aligner design, consistent with previous clinical evaluations that quantify planned movement as the difference between the digital setup and the pretreatment tooth position^8^. In current clinical practice, setup distance is largely determined empirically, and its biomechanical implications are not yet fully understood^9^.

Excessive orthodontic force has been shown to be ineffective in accelerating tooth movement^10^ and may instead induce periodontal ligament (PDL) damage^11^, tissue hyalinization^12^, root resorption^13,14^. Previous biological and experimental studies suggest that efficient orthodontic tooth movement occurs when compressive loading of the PDL remains within a physiological range, promoting osteoclast recruitment and bone resorption at compression sites while avoiding extensive ischemic injury and hyalinization^15,16^. This physiological pressure range has been suggested to lie between capillary pressure (approximately 4.7 kPa) and systolic pressure (approximately 16 kPa)^15^.

Although the biomechanical principles governing orthodontic tooth movement have been extensively investigated in conventional fixed appliance systems^17^, their direct application to clear aligner therapy remains limited, and the underlying force systems are not yet fully characterized^18,19^. Most finite element analysis (FEA) studies on clear aligners have focused on aligner geometry, attachment design, or displacement outcomes, while systematic reviews highlight a lack of direct evaluation of PDL hydrostatic pressure and its implications for alveolar bone remodeling, particularly with respect to setup distance selection^9,18,20^.

Maxillary canine retraction plays a central role in extraction-based orthodontic treatment, as it largely determines subsequent space-closure mechanics and anchorage control. Clinical studies have shown that canine movement in clear aligner therapy is among the least predictable, with frequent uncontrolled tipping and discrepancies between planned and achieved root movement, highlighting the need for biomechanically informed setup design^5,6,19^.

Therefore, the purpose of this study was to establish a biomechanical framework for evaluating clear aligner setup distance based on PDL pressure distribution. Using three-dimensional finite element analysis, upper canine retraction was simulated across a range of setup distances for both bodily and tipping movement designs. By quantifying the spatial distribution of hydrostatic pressure within the PDL and evaluating the proportion of tissue subjected to physiologically relevant pressure conditions, this study aims to clarify how setup distance influences the mechanical environment of the PDL and to propose rational biomechanical criteria for clear aligner setup design.

## Materials and methods

**Fig. 1:**
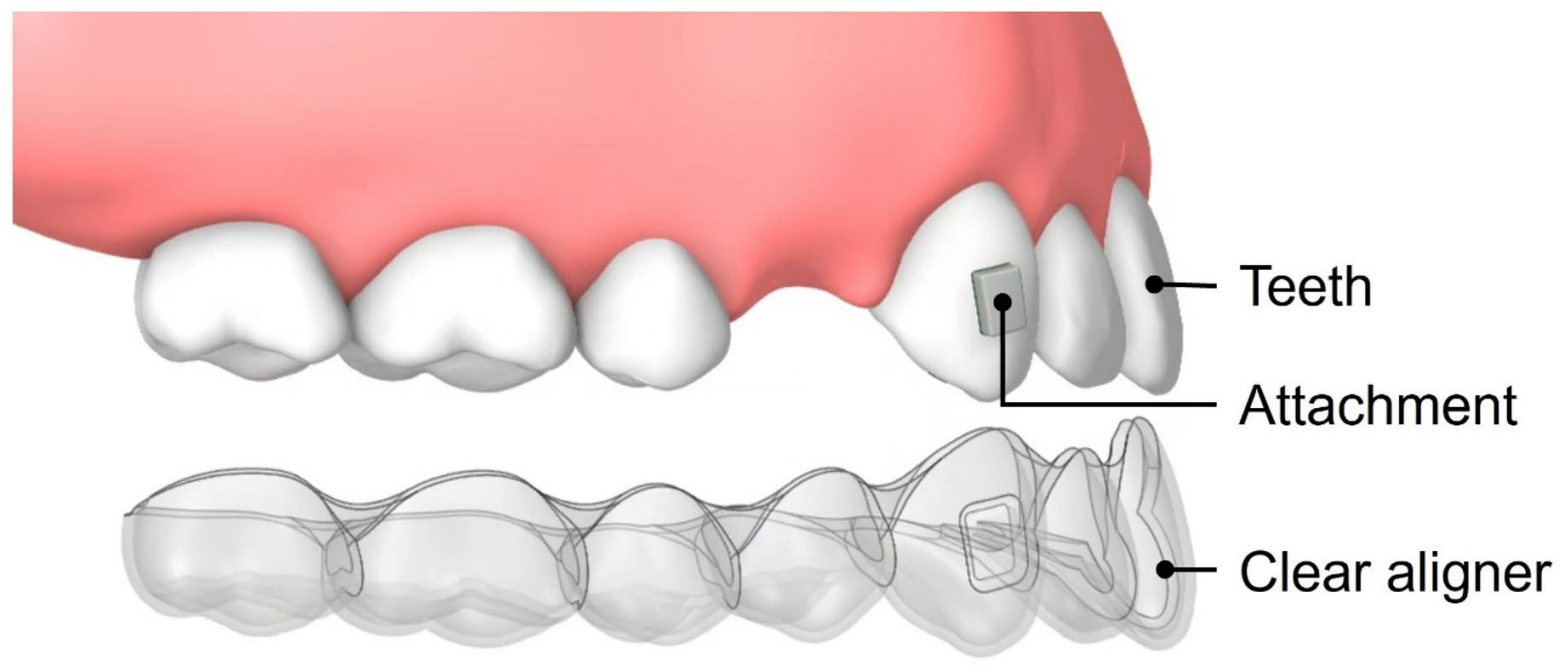
Three-dimensional finite element model of the dentition and clear aligner system used in this study. The clear aligner is uniformly offset from the crown surface, and a vertical rectangular composite attachment is bonded to the labial surface of the maxillary canine to facilitate controlled tooth movement.

A three-dimensional FEA was performed using ANSYS Mechanical 2024R (ANSYS Inc., Canonsburg, PA, USA) to simulate upper canine retraction for space closure. Clear aligners were modeled with an approximate thickness of 0.5 mm and positioned with a uniform offset from the crown surface (Fig. 1). A vertical rectangular composite attachment measuring 2 mm × 3 mm × 1 mm was bonded to the labial surface of the canine. The periodontal ligament (PDL) was modeled with a uniform thickness of 0.25 mm and offset from the root surface.

The material properties of the teeth, clear aligner, attachment, and PDL were assigned based on previously published studies^21,22^. A bilinear elastic material model was applied to the PDL, consisting of an initial elastic modulus ($\:{E}_{1}$) followed by a secondary modulus ($\:{E}_{2}$) after the transition strain ($\:{\epsilon\:}_{12}$). The material properties used in the analysis are summarized in Table 1.

**Table 1:** Material properties assigned to the teeth, clear aligner, attachment, and PDL in the finite element model. The PDL was modeled using a bilinear elastic material with an initial modulus ($\:{E}_{1}$), a secondary modulus ($\:{E}_{2}$), and a transition strain ($\:{\epsilon\:}_{12}$).

	Young’s modulus (MPa)	Poisson’s ratio
Teeth	1.96 × 10^4^	0.30
Clear aligner	528	0.36
Attachment	12.5 × 10^3^	0.36
PDL	Bilinear $\:{E}_{1}=0.05,\:{E}_{2}=0.20,\:{\epsilon\:}_{12}=0.07$	0.3

To induce tipping movement, a conventional clear aligner design was used in which the canine was positioned in a retracted configuration without rotational compensation. In contrast, bodily movement was achieved by implementing a previously described compensation protocol designed to counteract the inherent tipping tendency of aligner-induced tooth movement^23^. The anti-tipping compensation rotation was implemented as a geometric design operation about an axis passing through the crown center of mass of the canine, providing a consistent and reproducible reference for all setup configurations.

Setup distances were varied from 0.05 mm to 0.35 mm in increments of 0.05 mm to evaluate their influence on force transmission and PDL pressure distribution.

The compensation protocol iteratively reduced the tipping angle by applying the resultant tipping angle in the opposite rotational direction to the canine segment of the clear aligner. The initial tipping angles associated with tipping movement and the residual tipping angles after compensation for bodily movement are listed in Table 2. The results demonstrate that tipping angles were substantially reduced after applying the compensation protocol, indicating successful induction of bodily movement.

**Table 2:** Tipping angles of the canine for tipping and bodily movement designs across different setup distances, and corresponding anti-tipping angles incorporated into the clear aligner to induce bodily movement through the compensation protocol.

Setup distance (mm)	Tipping angle of tipping movement (deg)	Tipping angle of Bodily movement (deg)	Anti-tipping angle (deg)
0.05	0.197443	0.074584	0.3932
0.10	0.302329	0.085454	0.7727
0.15	0.450407	0.119737	1.1226
0.20	0.568347	0.146245	1.6499
0.25	0.684928	0.177679	2.0568
0.30	0.822338	0.181173	4.0852
0.35	0.951738	0.188313	4.7141

FEA, finite element analysis; PDL, periodontal ligament.

The anti-tipping angles incorporated into the clear aligner design to achieve bodily movement are also presented in Table 2. Larger anti-tipping angles were required at greater setup distances, reflecting the increased tipping tendency generated by larger aligner displacements.

**Fig. 2:**
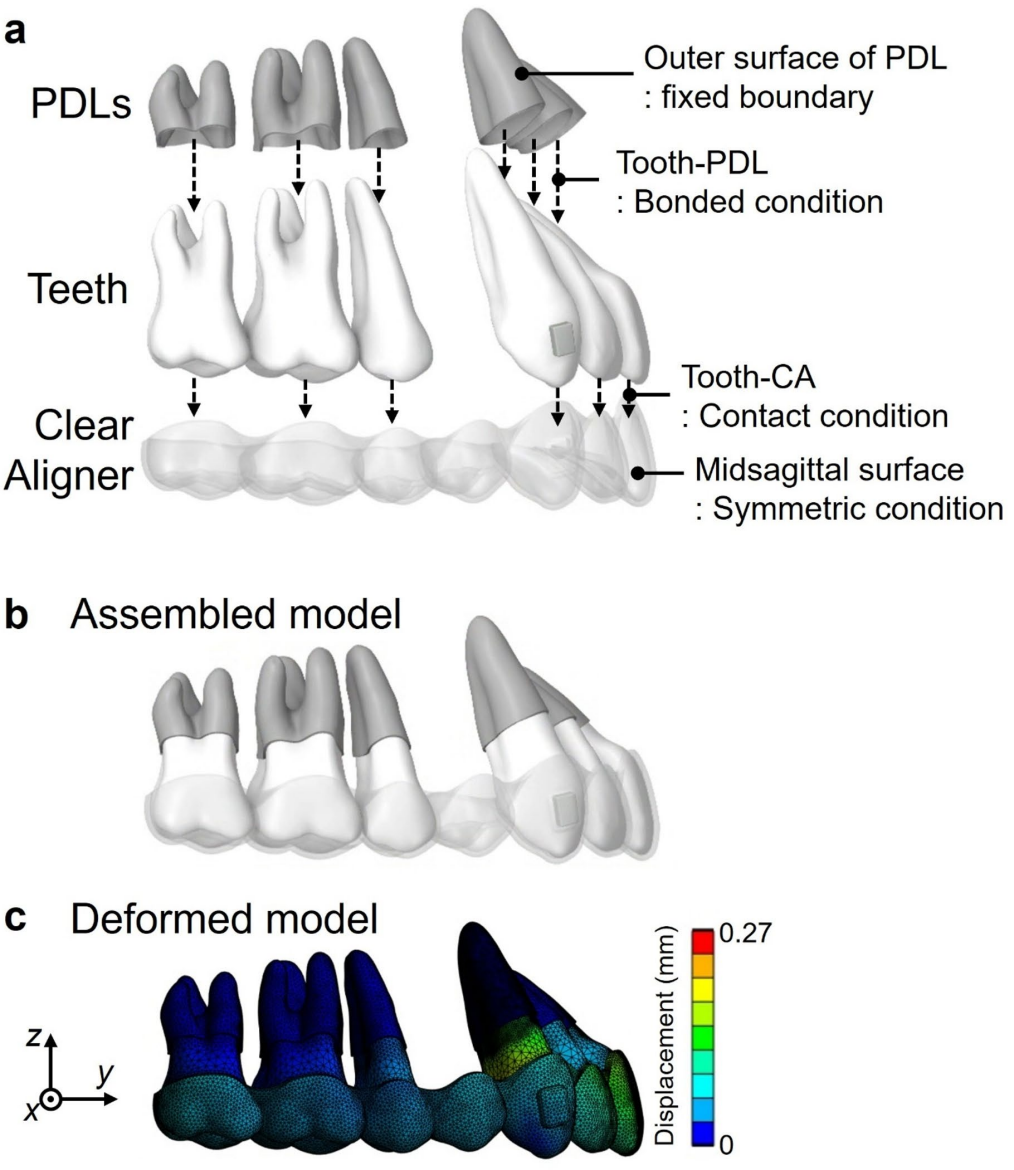
Finite element analysis setup for simulating upper canine retraction. (**a**) Components of the model, including teeth, periodontal ligament (PDL), alveolar bone, clear aligner, and applied boundary conditions. (**b**) Assembled finite element model before deformation. (**c**) Deformed configuration after application of the setup distance, illustrating the mechanical equilibrium of the system.

The schematic of the finite element model is shown in Fig. 2(a) and (b). Because the maxilla is significantly stiffer than the surrounding PDL, the outer surfaces of the PDL were constrained using fixed boundary conditions. Bonded contact conditions were defined between the teeth and the inner surfaces of the PDL, while contact interactions were defined between the teeth (including the attachment) and the clear aligner. Contact interactions between the clear aligner and the tooth–attachment complex were modeled using an augmented Lagrange formulation with a friction coefficient of 0.2. Automatic normal stiffness and program-controlled penetration tolerance were employed. These settings were selected to ensure numerical stability and consistency with previous aligner FEA studies^22,23^. To reduce computational complexity, symmetry was assumed along the midsagittal plane.

The finite element model consisted of approximately 535,733 elements and 121,346 nodes. A formal mesh convergence study was not performed in the present analysis. Although mesh refinement was applied in the periodontal ligament region to capture stress gradients, further verification through convergence testing would strengthen the robustness of quantitative results. However, as the present study focuses on comparative trends under consistent modeling conditions, the relative differences between tipping and bodily movement are expected to be less sensitive to mesh discretization.

**Fig. 3:**
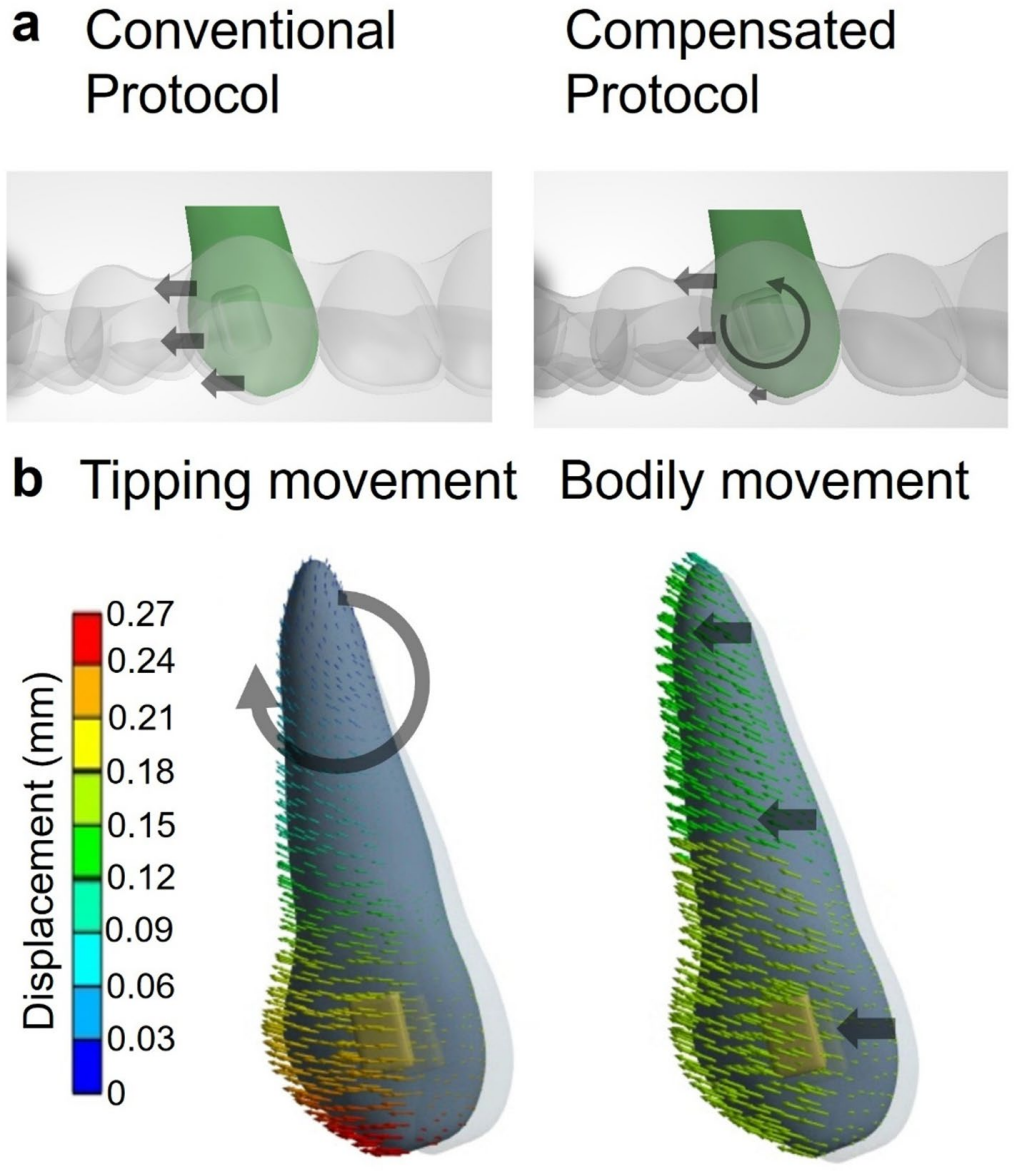
(**a**) The design scheme of the clear aligner of both conventional and compensated protocols. The clear aligner in conventional protocol is purely retracted, whereas that in compensated protocol is rotated along the center of the mass of crown part to counteract tipping movement. (**b**) Displacement patterns of the maxillary canine under a setup distance of 0.25 mm for tipping and bodily movement designs. Differences in displacement magnitude and direction reflect distinct movement kinematics.

The geometric mismatch between the canine and the clear aligner introduced by the setup distance was resolved through mechanical equilibrium, resulting in overall deformation and stress development within the system (Fig. 2(c)). To more precisely distinguish tipping and bodily movement conditions in the present model, two different aligner design protocols were implemented (Fig. 3a). In the non-compensated protocol, the canine was displaced according to the prescribed setup distance without rotational compensation, resulting in an uncontrolled tipping tendency due to asymmetric force application relative to the center of resistance. In the compensated protocol, an anti-tipping rotational component was incorporated into the aligner design to counteract the moment generated by distal displacement, thereby promoting translational (bodily) movement.

The distinction between tipping and bodily movement was evaluated based on the spatial distribution and directionality of displacement vectors along the root surface (Fig. 3b). Tipping movement was characterized by a rotational displacement pattern with greater cervical displacement and an apically located center of rotation. In contrast, bodily movement exhibited near-parallel displacement vectors along the long axis of the tooth, indicating reduced rotational tendency and more uniform root translation.

To quantitatively characterize the mechanical environment within the PDL, pressure metrics were extracted, including maximum compressive pressure, maximum tensile pressure, 95th percentile pressure, and the coefficient of variation (CV) (Supplementary Tables S1 and S2). For statistical summaries of hydrostatic pressure, element area-weighted quantities were computed to reduce mesh-density bias. Each elemental pressure value $\:{p}_{i}$ was weighted by its corresponding PDL surface element area $\:{w}_{i}$. The area-weighted mean and standard deviation were calculated as

1 $$\:{\mu\:}_{w}=\frac{{\sum\:}_{i}{w}_{i}{p}_{i}}{{\sum\:}_{i}{w}_{i}}$$

and

2 $$\:{\sigma\:}_{w}=\sqrt{\frac{{\sum\:}_{i}{w}_{i}{\left({p}_{i}-{\mu\:}_{w}\right)}^{2}}{{\sum\:}_{i}{w}_{i}}}$$

respectively. Pressure uniformity was quantified using the CV calculated separately for compressive ($\:{p}_{i}<0$) and tensile ($\:{p}_{i}>0$) pressure domains. Because the pressure field was evaluated over the entire computational domain rather than sampled from a statistical population, variance measures were computed without Bessel correction. In addition, the fraction of PDL area within the physiological range (between 4.7 and 16 kPa) and exceeding the suprasystolic pressure threshold (16 kPa) was calculated to evaluate the extent of potentially excessive mechanical loading.

## Results

### Force and moment generation as a function of setup distance

**Fig. 4:**
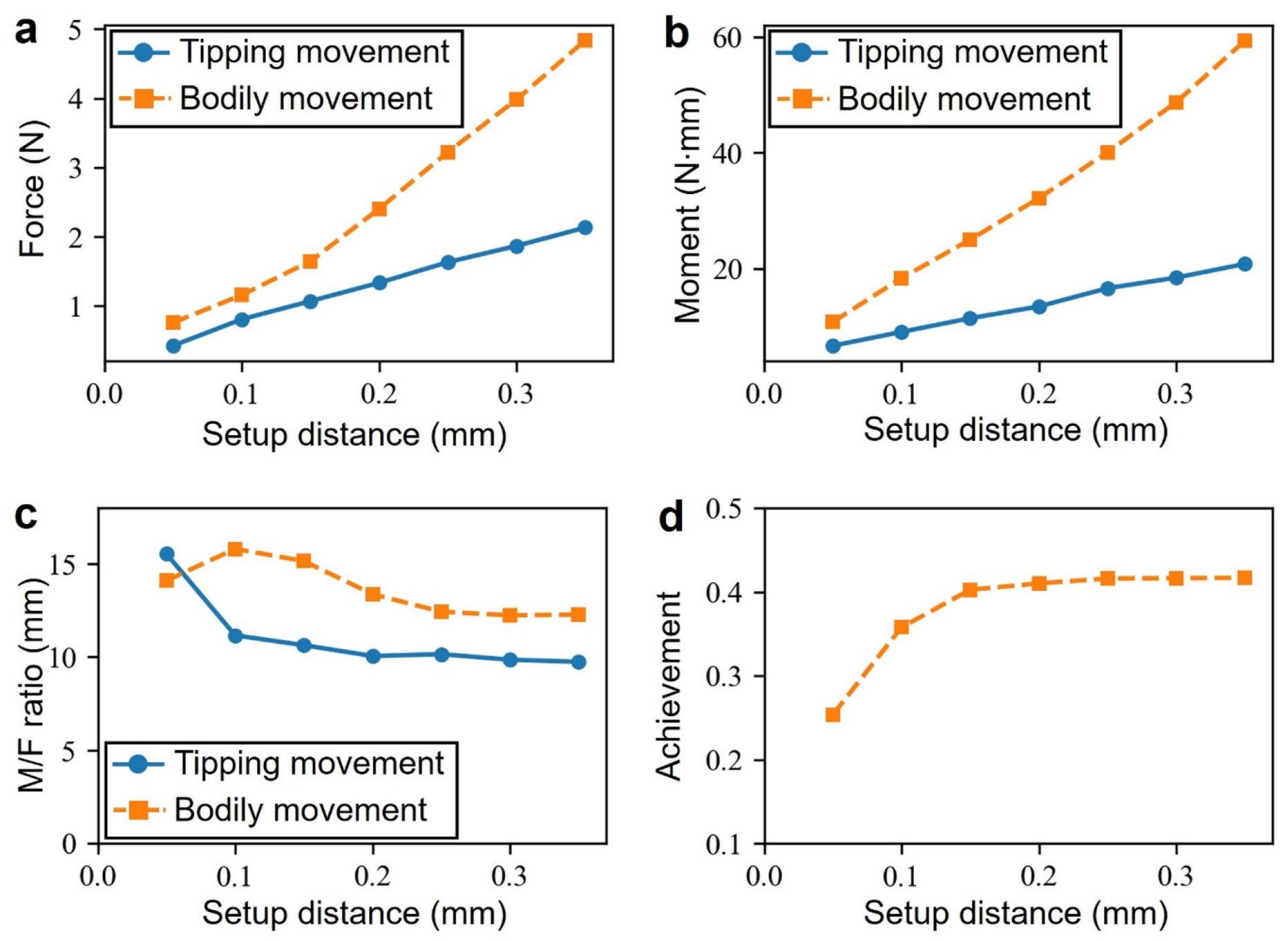
Contact forces and moments acting between the maxillary canine and the clear aligner as a function of setup distance for tipping and bodily movements. (**a**) Resultant force magnitude. (**b**) Resultant moment magnitude. (**c**) Moment to force ratio. (**d**) Achievement of bodily movement, defined as the ratio of actual displacement to planned displacement with respect to setup distance.

Figures 4(a and b) illustrate the forces and moments acting between the maxillary canine and the clear aligner as a function of setup distance. As the setup distance increased, both the contact forces and the moments increased for tipping and bodily movement designs. Notably, bodily movement generated larger forces and moments than tipping movement at the same setup distance, with larger moment magnitudes observed in bodily movement compared with tipping movement at the same setup distance. The resultant force increased approximately linearly with setup distance, with an average slope of 0.28 N per 0.05 mm for tipping and 0.68 N per 0.05 mm for bodily movement. Correspondingly, the resultant moment increased by 2.36 N·mm per 0.05 mm for tipping and 8.10 N·mm per 0.05 mm for bodily movement.

Figure 4(c) shows the moment to force ratio (M/F ratio) of both movements. At the smallest setup distance (0.05 mm), the M/F ratio was slightly higher in tipping movement than in bodily movement. This difference diminished with increasing setup distance, after which bodily movement exhibited consistently larger M/F ratios.

### Displacement characteristics of tipping and bodily movement

These displacement characteristics correspond to differences in force and moment magnitudes between tipping and bodily movement conditions described in Sect. 3.1.

The achievement of bodily movement, defined as the ratio of actual tooth movement to the planned movement with respect to the setup distance, was calculated following a previously reported method^23^. The achievement was defined as Eq. (3):

3 $$\:achievement=1-\frac{\sum\:\left|\overrightarrow{D}-\overrightarrow{r}\right|\varDelta\:A}{\left|\overrightarrow{D}\right|\sum\:\varDelta\:A}$$

where $\:\left|\overrightarrow{D}\right|$ denotes the setup distance, $\:\overrightarrow{r}$ is the displacement vector of each finite element, and $\:\varDelta\:A$ is the corresponding element area. The achievement values for bodily movement at different setup distances are presented in Fig. 4(d). As the setup distance increased, the achievement value increased and gradually converged to approximately 41%.

Achievement was defined as a geometric measure of deviation from the prescribed setup target rather than the magnitude of displacement along the setup direction. Accordingly, achievement reflects the extent to which the resulting tooth movement remains aligned with the intended movement objective.

Although bodily setup was prescribed along a defined translational direction, the resulting tooth displacement did not occur strictly along the setup vector. Instead, displacement deviated by approximately 32° due to the combined force–moment system generated during aligner engagement. Because achievement quantifies directional agreement with the prescribed setup objective rather than displacement magnitude alone, such angular deviation leads to lower achievement values despite comparable displacement magnitude. A geometric consistency check confirmed that the measured displacement magnitude and angular deviation produce achievement values consistent with those shown in Fig. 4(d), indicating that the apparent discrepancy reflects directional misalignment of tooth movement rather than reduced mechanical response.

### Hydrostatic pressure distribution within the periodontal ligament

**Fig. 5:**
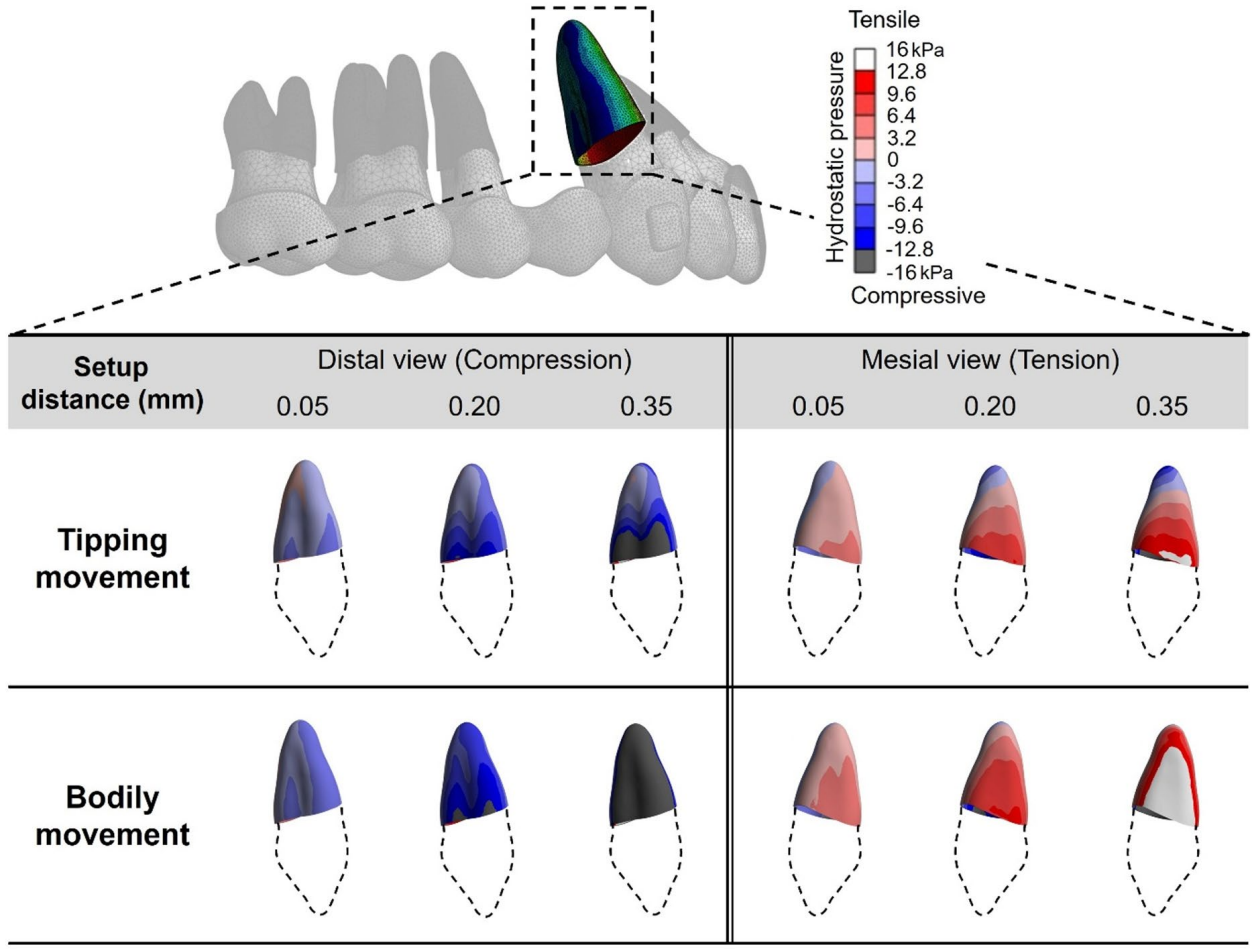
Spatial distribution of hydrostatic pressure within the PDL of the maxillary canine across different setup distances for tipping and bodily movements. Distal views indicate compressive regions, whereas mesial views indicate tensile regions, highlighting movement-dependent pressure patterns.

The hydrostatic pressure in the PDL, denoted as $\:{\sigma\:}_{H}$, was defined as one third of the trace of the stress tensor, as expressed in Eq. (4):

4 $$\:{\sigma\:}_{H}=({\sigma\:}_{xx}+{\sigma\:}_{yy}+{\sigma\:}_{zz})/3\:$$

where $\:{\sigma\:}_{xx}$, $\:{\sigma\:}_{yy}$ and $\:{\sigma\:}_{zz}$ represent the normal stresses components in the Cartesian coordinate system. Figure 5 illustrates the distribution of hydrostatic pressure within the PDL of the canine across different setup distances for both tipping and bodily movements. Due to the retractive force applied to the canine, compressive stresses are primarily observed on the distal surfaces, whereas tensile stresses appeared on the mesial surfaces. The negative hydrostatic pressure corresponds to compression and positive values indicate tension.

As the setup distance increased, the overall magnitude of hydrostatic pressure within the PDL increased for both movement patterns. Larger setup distances displaced the equilibrium position of the canine–aligner system further in the retraction direction, leading to higher force levels within the system. Consequently, higher stress and pressure levels were observed in both compressive and tensile regions of the PDL.

In tipping movement, the cervical region of the PDL consistently exhibited a substantially higher hydrostatic pressure magnitude than the apical region. In contrast, bodily movement showed a relatively uniform pressure distribution throughout the entire PDL. This difference in spatial pressure distribution was observed across all examined setup distances, indicating a clear distinction between the two movement patterns.

Quantitative pressure metrics summarized in Supplementary Tables S1 and S2 demonstrated consistent increases in both compressive and tensile pressure magnitudes with increasing setup distance. Bodily movement generated higher maximum and 95th percentile pressures compared with tipping movement at equivalent setup distances, with higher overall force levels observed.

Despite higher pressure magnitudes, bodily movement maintained substantially lower coefficients of variation for both compressive and tensile pressures, with lower spatial variability in pressure distribution. In contrast, tipping movement exhibited progressively increasing CV values with setup distance, reflecting increasing pressure localization.

### Optimal-pressure fraction and identification of effective setup-distance range

**Fig. 6:**
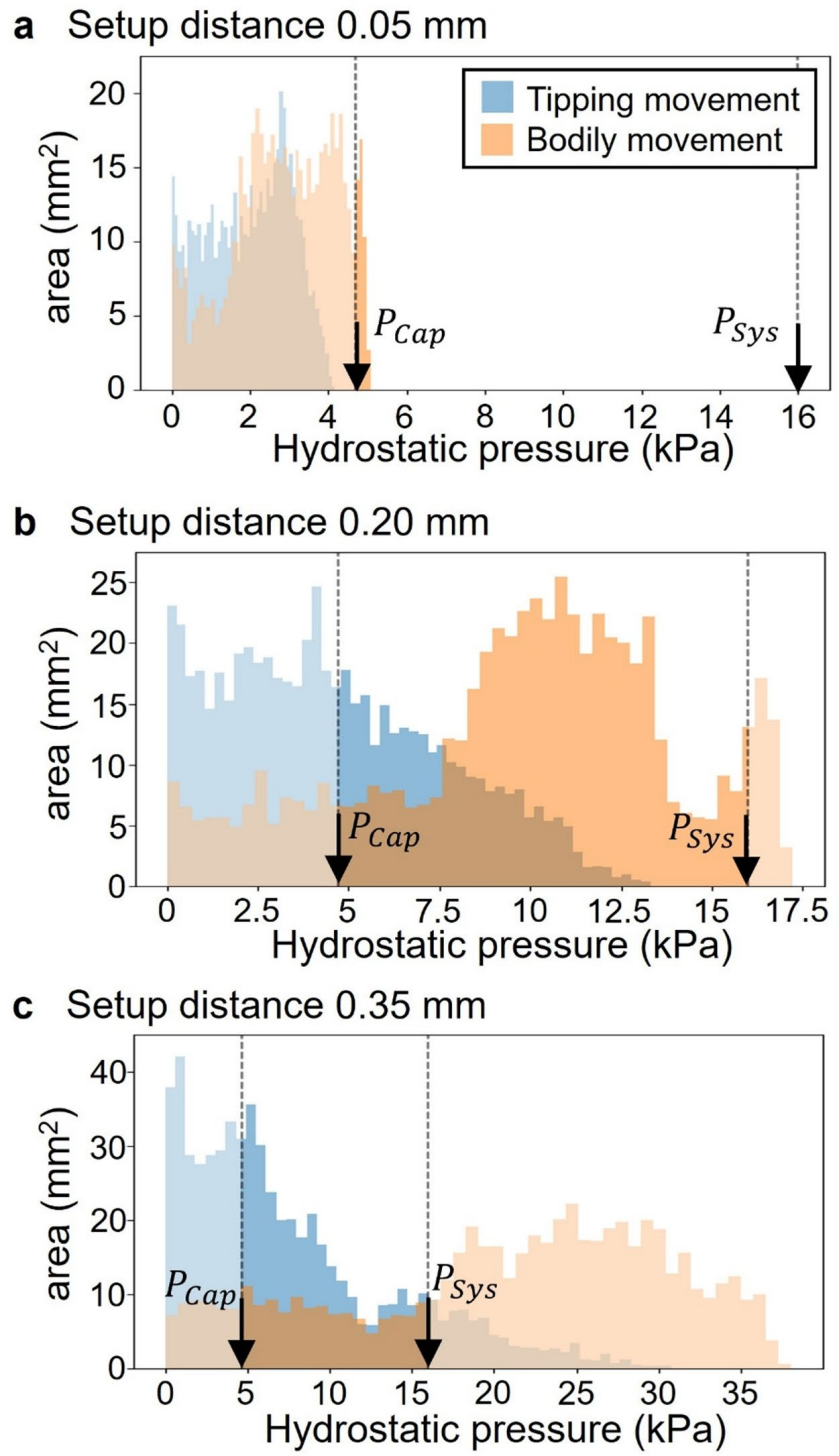
Histograms of hydrostatic pressure distribution within the PDL of the canine for setup distances of (**a**) 0.05 mm, (**b**) 0.20 mm, and (**c**) 0.35 mm. Results for both tipping and bodily movements are shown. Dashed lines denote the capillary pressure ($\:{P}_{cap}$) and systolic pressure ($\:{P}_{sys}$), defining the limits for the physiological pressure range for effective bone remodeling.

To characterize the spatial distribution of PDL pressure, histograms of hydrostatic pressure were generated for each setup distance. Figure 6 presents the histograms for both tipping and bodily movements, illustrating the proportion of PDL area corresponding to infracapillary, optimal physiological, and suprasystolic pressure ranges. As the setup distance increased, the fraction of PDL within the optimal pressure range initially increased and subsequently decreased due to the expansion of the suprasystolic pressure region. This trend was observed for both movement patterns, although the effective pressure range differed between tipping and bodily movements.

## Discussion

The present study establishes a biomechanical relationship between clear aligner setup distance and the spatial distribution of periodontal ligament pressure under tipping and bodily movement conditions.

Tooth movement was induced through prescribed geometric displacement rather than externally applied orthodontic forces, enabling assessment of the mechanical response generated directly by aligner–tooth interaction. The lower M/F value observed in bodily movement (Fig. 4c) at the smallest setup distance corresponds to limited aligner deformation and reduced engagement of the compensatory rotational component under minimal geometric mismatch. The observed transition in M/F ratio with increasing setup distance suggests that effective transmission of compensatory moments depends on sufficient aligner deformation and contact engagement.

Because tooth movement results from mechanical equilibrium under complex force–moment interactions, displacement may occur along directions deviating from the prescribed setup vector, leading to differences between displacement magnitude (Fig. 3b) and achievement-based metrics (Fig. 4d).

Under tipping conditions, the center of rotation was located near the root apex, resulting in a displacement pattern dominated by rotational motion (Fig. 3b). The force generated by the setup distance is transmitted across the crown through aligner contact, producing a moment about the center of rotation. Consequently, due to the lever-arm effect, points farther from the center of rotation—such as the cusp tip—exhibit larger displacement magnitudes. Thus, the relatively large displacement observed at the crown does not indicate negligible aligner deformation, but instead reflects rotation-dominated tooth movement.

A compensation angle capable of minimizing tipping toward zero may theoretically exist for a given setup condition. However, due to the nonlinear relationship between aligner deformation, contact interaction, and force–moment generation, such a configuration cannot be determined analytically and would require iterative numerical exploration. The present compensation strategy therefore represents a progressive geometric adjustment that decreases tipping angle rather than an optimization procedure intended to eliminate tipping completely.

As setup distance increases, larger compensatory rotations are required to counteract tipping tendency. When the compensation rotation is applied about the crown center of mass, this geometrically induces amplified displacement at the root region. Consequently, localized deformation within the periodontal ligament increases substantially, producing elevated strain levels and strongly nonlinear mechanical responses. Under such conditions, stable force–moment equilibrium becomes increasingly difficult to achieve, which limits the effectiveness of rotational compensation at larger setup distances.

The pressure gradient observed in tipping movement (Fig. 5) can be attributed to its inherent rotational kinematics. During tipping, the tooth rotates about a center of rotation located near the apical region of the root^24^ as shown in Fig. 3(b), which results in greater displacement and deformation in the cervical region compared with the apical region. Consequently, stress and hydrostatic pressure are concentrated cervically, giving rise to a pronounced cervico-apical pressure gradient within the PDL.

At small setup distances (0.05 mm), most of the PDL remains below the capillary pressure threshold, indicating insufficient mechanical stimulus for effective tooth movement. Although increasing setup distance elevates the overall force level, pressure escalation occurs predominantly in the cervical region due to rotational motion, often exceeding the systolic pressure threshold while the apical region remains underloaded. As a result, the area subjected to physiologically optimal pressure is confined to a limited mid-root region, inherently restricting both efficiency and biological safety of tipping-dominated movement.

In contrast, bodily movement produces near-parallel translation of the tooth, leading to relatively uniform displacement along the root surface and a homogeneous PDL pressure distribution. As the setup distance increases, a progressively larger portion of the PDL enters the physiological pressure range without excessive localized pressure, allowing a broad effective setup distance range. Suprasystolic pressure emerges more uniformly only at larger setup distances, enabling bodily movement to maintain a wide range of setup distances in which a substantial area of the PDL remains within the optimal pressure zone.

Because orthodontic tooth movement arises from spatially heterogeneous tissue responses rather than isolated stress concentrations, evaluating orthodontic biomechanics solely based on local extreme values, such as peak stress or pressure^25^, may not adequately represent the overall mechanical conditions associated with tooth movement. Conversely, averaging stress over the entire PDL^15^ can obscure clinically relevant spatial heterogeneity, such as the coexistence of infracapillary and suprasystolic regions. Therefore, histogram-based analysis (Fig. 6) was adopted to capture the spatial distribution of PDL pressure relative to biologically meaningful thresholds.

**Fig. 7:**
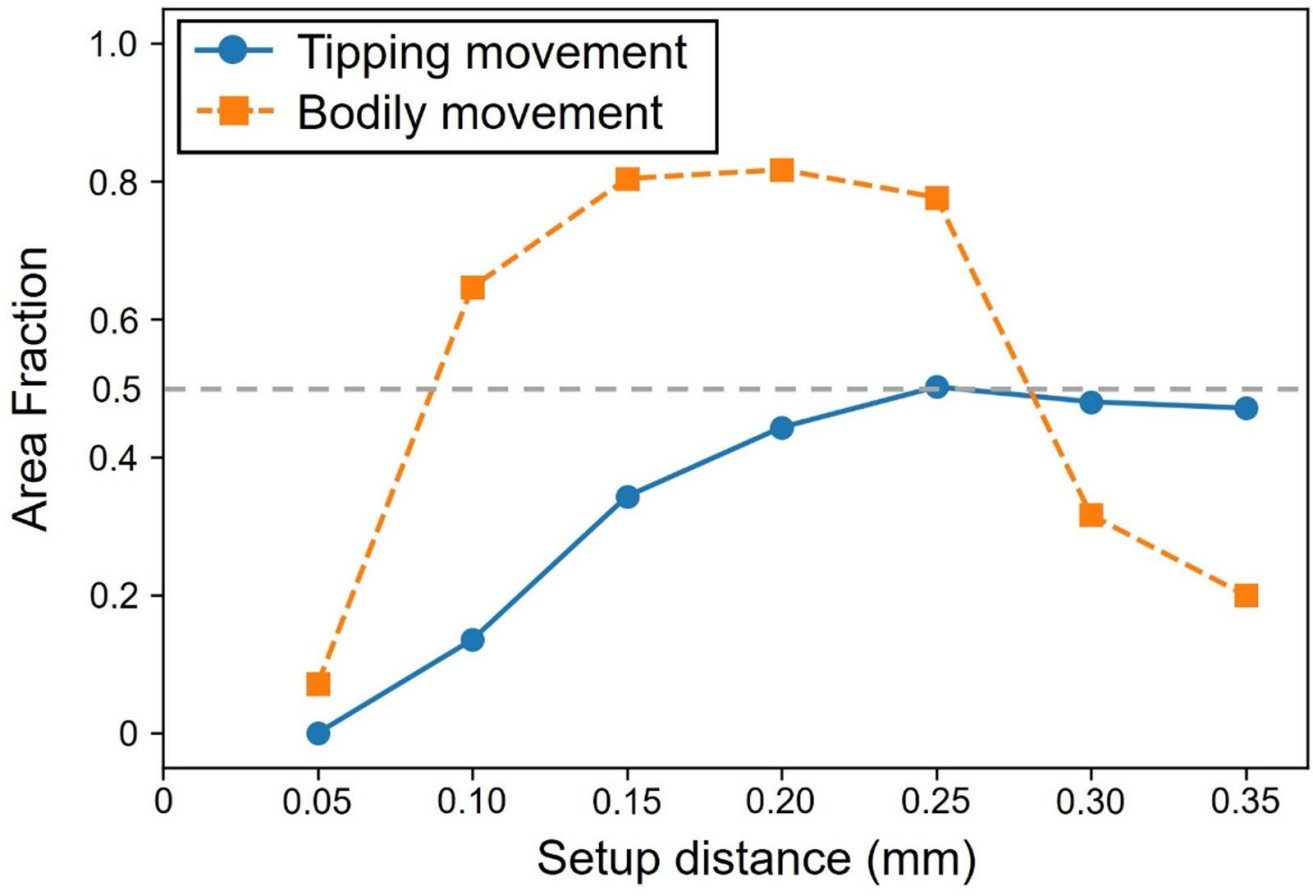
Area fraction of the periodontal ligament subjected to hydrostatic pressure within the physiological range as a function of setup distance for tipping and bodily movements.

The area fraction within the optimal pressure range represents the spatial proportion of the PDL subjected to hydrostatic pressure levels associated with physiologically relevant remodeling conditions (Fig. 7). This metric is defined as a deterministic area ratio rather than a probabilistic measure and quantifies the extent of tissue regions experiencing pressure within the predefined physiological window. Higher values therefore indicate broader spatial engagement of the PDL within pressure conditions considered favorable for orthodontic tooth movement. Importantly, this metric should be interpreted as a biomechanical surrogate indicator rather than a direct predictor of biological remodeling outcomes, as biological responses depend on additional cellular and biochemical processes not captured in the present model.

The optimal-pressure area fraction initially increased with setup distance as a progressively larger portion of the PDL entered the physiological pressure range between capillary and systolic thresholds. Quantitative analysis (Supplementary Table S1) showed that, within intermediate setup distances (approximately 0.15–0.25 mm), increases in pressure magnitude primarily shifted previously underloaded regions into the optimal range while suprasystolic areas remained limited.

Beyond this range, further increases in setup distance resulted in rapid expansion of PDL regions exceeding the suprasystolic threshold, particularly in bodily movement. As demonstrated by the marked increase in suprasystolic area fraction at setup distances greater than 0.25 mm, elevated pressure levels increasingly displaced tissue regions beyond the physiological window, leading to a reduction in optimal-pressure fraction despite continued increases in overall pressure magnitude.

In bodily movement, the uniform pressure distribution allows a wide range of setup distances (0.10–0.25 mm) to maintain a large optimal pressure area, providing substantial tolerance to design variability. In contrast, tipping movement inherently produces a cervico-apical pressure gradient that constrains expansion of the optimal pressure region, rendering the movement biologically efficient and safe only within a narrow setup distance range centered around 0.25 mm. These findings indicate that the biological response to clear aligner-induced tooth movement is strongly dependent on the type of movement.

Although the setup distance corresponding to the maximum area fraction within the physiological pressure range is smaller for bodily movement than for tipping movement, this difference can be attributed to the greater force and moment generated by bodily movement at the same setup distance (Fig. 4). Consequently, the setup distance required to achieve widespread engagement of physiologically relevant pressure conditions is reduced in bodily movement compared with tipping movement. As setup distance increases, the area fraction within the physiological pressure range in bodily movement approaches a plateau, indicating that setup distances of approximately 0.25 mm produce extensive spatial engagement of the PDL within the optimal pressure window. However, larger setup distances require greater anti-tipping rotation (Table 2), potentially increasing geometric mismatch between the aligner and dentition and thereby compromising aligner fit and mechanical predictability.

These findings suggest that clear aligner setup distance should be determined based on biomechanical criteria rather than empirical convention. Bodily movement permits a wider tolerance range in which a substantial portion of the PDL remains within the physiological pressure window. In contrast, tipping movement exhibits a narrow effective range, as localized pressure escalation limits expansion of the optimal pressure zone and increases the risk of adverse tissue responses.

In conventional orthodontic treatment, tooth movement often proceeds through an initial tipping phase followed by a finishing stage involving uprighting and root correction to achieve proper axial alignment^26^. This finishing phase, which biomechanically resembles tipping movement, is associated with concentrated stress within the PDL and increased biological burden on the supporting tissues^27^. From this perspective, designing tooth movement to approximate bodily translation from the outset may reduce the need for extensive uprighting procedures and promote more efficient and biologically favorable treatment progression.

When tipping movement is clinically unavoidable, the results indicate that it should be implemented cautiously using small incremental tipping angles. Rather than inducing pronounced tipping within a single phase, embedding limited tipping components within predominantly bodily movement across multiple stages may help prevent excessive localized pressure, minimize the risk of hyalinization, and enable gradual, controlled root movement. The broader tolerance associated with bodily movement allows minor tipping components to be accommodated within the physiological pressure range without compromising treatment efficiency. Such staged biomechanics are consistent with established orthodontic principles emphasizing controlled force systems and progressive root movement to achieve stable long-term outcomes^26,27^.

The elastic modulus assigned to the aligner reflects thermoformed material behavior under intraoral conditions. Previous studies have demonstrated that thermoforming and intraoral aging substantially alter the mechanical properties of thermoplastic aligner materials compared with raw sheet properties^28,29^. Accordingly, the modulus used in the present study represents clinically relevant material behavior rather than room-temperature sheet stiffness. In addition to material stiffness, aligner thickness represents another key determinant of appliance rigidity and force transmission, and variations in thickness may similarly influence the resulting pressure distribution and effective setup distance.

Several limitations of the present study should be acknowledged. The periodontal ligament was modeled using a bilinear elastic formulation that does not capture its viscoelastic behavior, anisotropy, or inter-individual variability, which may influence absolute stress magnitudes. Although this approach is commonly employed in finite element simulations, numerical sensitivity may arise near the transition strain. The present analysis focuses on sustained loading conditions rather than transient viscoelastic responses immediately after aligner engagement. While viscoelasticity may influence time-dependent stress relaxation, its incorporation in future models would allow a more comprehensive representation of the mechanical environment during prolonged aligner wear. In addition, the analysis was based on a single patient-specific anatomical model and an isolated canine movement scenario; thus, variations in root morphology, alveolar bone geometry, and PDL characteristics may affect the quantitative outcomes.

Furthermore, the predicted pressure distribution and effective setup-distance range are influenced by aligner mechanical properties, including material stiffness and thickness. Increased stiffness or thickness would lead to higher force transmission for a given setup distance, potentially shifting the optimal-pressure range toward smaller values, whereas more compliant aligners may require larger setup distances to achieve comparable pressure levels. It should be explicitly emphasized that the key quantitative findings of the present study—including the optimal setup-distance range of 0.10–0.25 mm for bodily movement and the narrower effective range centered around 0.25 mm for tipping movement—are strictly dependent on the specific material parameters and modeling assumptions adopted in this study. These findings may vary under different periodontal ligament properties (e.g., viscoelastic or hyperelastic constitutive models, alternative stiffness values), aligner characteristics (e.g., material modulus, thickness, geometry), or patient-specific anatomical conditions (e.g., root morphology, alveolar bone geometry, PDL thickness). Therefore, the numerical ranges reported herein should be interpreted as model-dependent biomechanical trends providing mechanistic insight rather than universally applicable clinical thresholds.

Moreover, the finite element model represents an idealized biomechanical environment and does not fully reproduce clinical complexity, including biological adaptation and time-dependent tissue remodeling. Consequently, the present findings should be interpreted as providing mechanistic insight into pressure distribution patterns associated with aligner-induced tooth movement rather than direct clinical predictions or universally applicable treatment recommendations.

Extension of the present framework to full-arch simulations incorporating multi-tooth movement and anchorage interactions represents an important direction for future investigation, since arch-level mechanical coupling may influence local force transmission and periodontal ligament loading patterns.

## Conclusion

This study proposes a biomechanical framework for evaluating clear aligner setup distance based on periodontal ligament pressure distribution rather than empirical convention. By quantifying the area fraction of the PDL subjected to physiologically relevant pressure levels, the present approach provides a spatially integrated descriptor of aligner-induced mechanical loading conditions. Finite element analyses of upper canine retraction demonstrated that bodily movement maintains a broader range of setup distances associated with relatively uniform pressure distribution, whereas tipping movement exhibits a narrower effective range due to localized pressure escalation and cervico–apical gradients. These findings indicate that bodily movement results in more uniform engagement of PDL pressure conditions across setup distances, whereas tipping-based strategies require stricter control to limit localized mechanical overloading. Clinically, the results suggest that treatment strategies emphasizing controlled bodily movement may help maintain favorable pressure distribution during space closure. Overall, pressure-based evaluation of setup distance provides a rational biomechanical framework for interpreting clear aligner setup design.

## Supplementary Information

Below is the link to the electronic supplementary material.

Supplementary Material 1

## Data Availability

All data associated with this study are presented in the paper.
