# Supplementary material for: Pressure-based finite element analysis of setup distance in clear aligner canine retraction comparing bodily and tipping movements
Source: Sci Rep. 2026 Apr 30;16:20198. doi: 10.1038/s41598-026-51497-9 (PMC13324254; doi:10.1038/s41598-026-51497-9)
Supplement: Supplementary file 1 — Supplementary Material 1 [file 41598_2026_51497_MOESM1_ESM.docx]

**Supplementary Materials**

**Pressure-Based Finite Element Analysis of Setup Distance in Clear Aligner Canine Retraction: Comparison of Bodily and Tipping Movements**

Kiyean Kim^1^, Youn-Kyung Choi^1,2^, Sung-Hun Kim^1^, Seong-Sik Kim^1^, Yong-Il Kim^1,2,*^

^1^Dental and Life Science Institute, School of Dentistry, Pusan National University, Yangsan, South Korea, 50612

^2^Department of orthodontics, dental Research Institute, School of Dentistry, Pusan National University, Yangsan, South Korea, 50612

**Supplementary Table S1**. Quantitative metrics of compressive hydrostatic pressure within the periodontal ligament (PDL) for tipping and bodily movement conditions across different setup distances. Reported values include maximum compressive pressure, 95th percentile pressure, area fraction within physiological range (between 4.7 kPa and 16 kPa), exceeding the suprasystolic threshold (16 kPa), and the coefficient of variation (CV) calculated using element area-weighted statistics. CV values represent spatial heterogeneity of compressive pressure distribution.

| Movement | Setup distance (mm) | Compressive pressure (kPa) | | | Area fraction (%) | |
| --- | --- | --- | --- | --- | --- | --- |
|  |  | Maximum | 95th percentile | Coefficient of variation | Between 4.7 and 16 kPa | exceeding 16 kPa |
| Tipping movement | 0.05 | 4.18 | 3.55 | 0.55 | 0 | 0 |
|  | 0.10 | 6.74 | 5.44 | 0.57 | 13.54 | 0 |
|  | 0.15 | 10.46 | 8.03 | 0.62 | 34.31 | 0 |
|  | 0.20 | 13.30 | 10.25 | 0.66 | 44.33 | 0 |
|  | 0.25 | 19.19 | 13.10 | 0.71 | 50.19 | 1.58 |
|  | 0.30 | 25.31 | 17.37 | 0.78 | 48.03 | 7.61 |
|  | 0.35 | 30.78 | 20.59 | 0.83 | 47.11 | 13.09 |
| Bodily movement | 0.05 | 5.10 | 4.79 | 0.45 | 7.10 | 0 |
|  | 0.10 | 9.72 | 8.62 | 0.43 | 64.64 | 0 |
|  | 0.15 | 11.75 | 11.20 | 0.40 | 80.37 | 0 |
|  | 0.20 | 17.23 | 16.32 | 0.43 | 81.68 | 6.85 |
|  | 0.25 | 21.56 | 20.38 | 0.45 | 77.66 | 28.89 |
|  | 0.30 | 27.72 | 25.86 | 0.45 | 31.61 | 50.20 |
|  | 0.35 | 38.09 | 34.58 | 0.47 | 19.97 | 71.25 |

**Supplementary Table S2**. Quantitative metrics of tensile hydrostatic pressure within the periodontal ligament (PDL) for tipping and bodily movement conditions across different setup distances. Reported values include maximum tensile pressure, 95th percentile pressure, and the coefficient of variation (CV) obtained from element area-weighted statistics, describing spatial variability of tensile loading within the PDL.

| Movement | Setup distance (mm) | Tensile pressure (kPa) | | |
| --- | --- | --- | --- | --- |
|  |  | Maximum | 95th percentile | Coefficient of variation |
| Tipping movement | 0.05 | 4.59 | 3.69 | 0.62 |
|  | 0.10 | 6.19 | 5.19 | 0.64 |
|  | 0.15 | 8.45 | 7.27 | 0.67 |
|  | 0.20 | 9.92 | 8.69 | 0.66 |
|  | 0.25 | 11.57 | 10.23 | 0.63 |
|  | 0.30 | 12.94 | 11.59 | 0.63 |
|  | 0.35 | 14.33 | 12.88 | 0.63 |
| Bodily movement | 0.05 | 4.96 | 4.02 | 0.48 |
|  | 0.10 | 7.20 | 6.03 | 0.47 |
|  | 0.15 | 9.48 | 8.16 | 0.46 |
|  | 0.20 | 11.32 | 9.90 | 0.45 |
|  | 0.25 | 13.03 | 11.60 | 0.44 |
|  | 0.30 | 14.84 | 13.28 | 0.43 |
|  | 0.35 | 16.73 | 15.05 | 0.43 |
